# Supplementary material for: Novel Driver Strength Index highlights important cancer genes in TCGA PanCanAtlas patients
Source: PeerJ. 2022 Aug 11;10:e13860. doi: 10.7717/peerj.13860 (PMC9375969; doi:10.7717/peerj.13860)
Supplement: Supplemental Information 2 [file peerj-10-13860-s002.zip › COHORTS/genes plots/cohorts/2021_8_16_14_9_distribution_cohorts_females_DLBC.pdf]

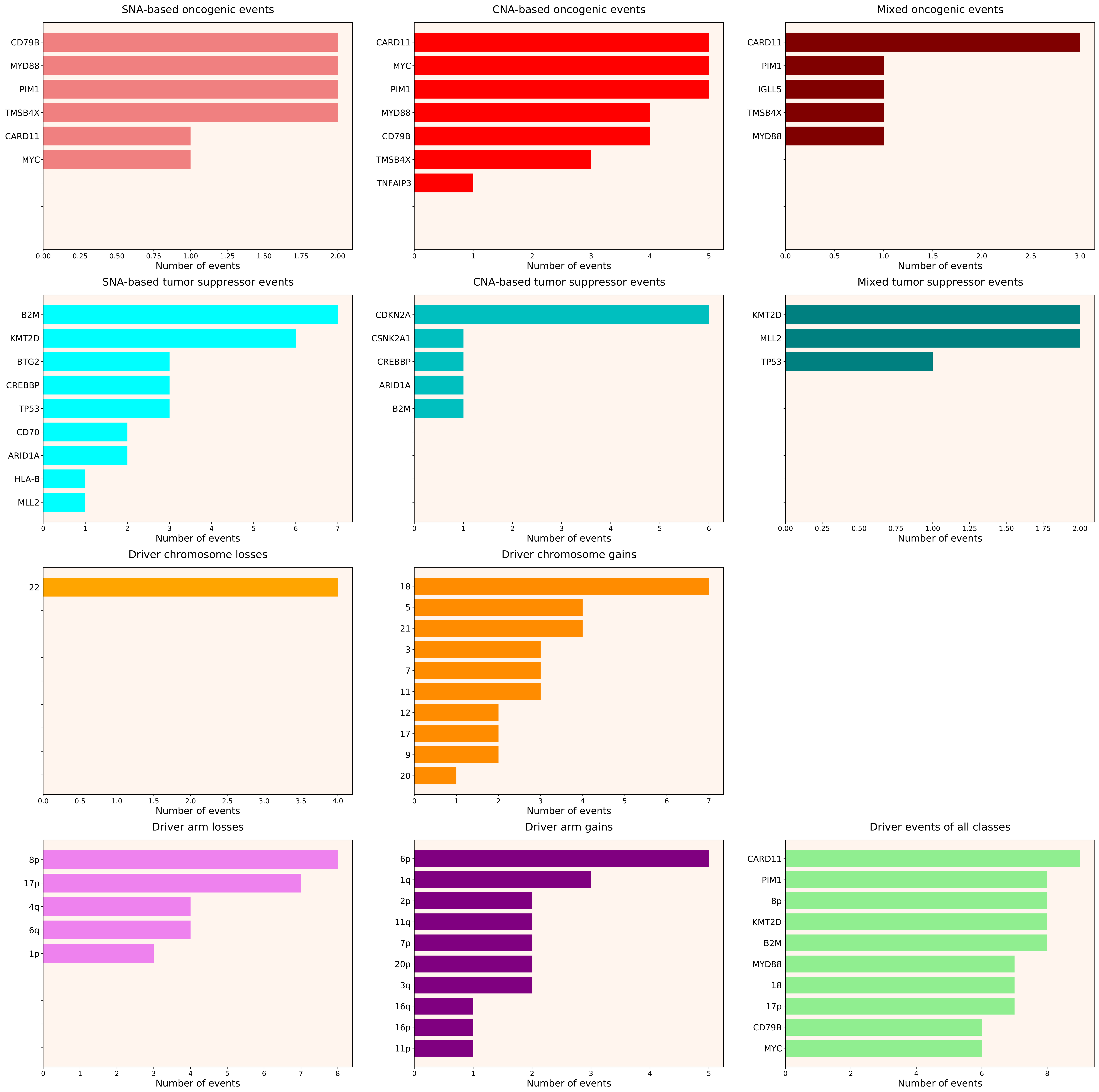
SNA-based oncogenic events
CD79B
MYD88
PIM1
TMSB4X
CARD11
MYC
0.00 0.25 0.50 0.75 1.00 1.25 1.50 1.75 2.00
Number of events
CNA-based oncogenic events
CARD11
MYC
PIM1
MYD88
CD79B
TMSB4X
TNFAIP3
0 1 2 3 4 5
Number of events
Mixed oncogenic events
CARD11
PIM1
IGLL5
TMSB4X
MYD88
0.0 0.5 1.0 1.5 2.0 2.5 3.0
Number of events
SNA-based tumor suppressor events
B2M
KMT2D
BTG2
CREBBP
TP53
CD70
ARID1A
HLA-B
MLL2
0 1 2 3 4 5 6 7
Number of events
CNA-based tumor suppressor events
CDKN2A
CSNK2A1
CREBBP
ARID1A
B2M
0 1 2 3 4 5 6
Number of events
Mixed tumor suppressor events
KMT2D
MLL2
TP53
0.00 0.25 0.50 0.75 1.00 1.25 1.50 1.75 2.00
Number of events
Driver chromosome losses
22
0.0 0.5 1.0 1.5 2.0 2.5 3.0 3.5 4.0
Number of events
Driver chromosome gains
18
5
21
3
7
11
12
17
9
20
0 1 2 3 4 5 6 7
Number of events
Driver arm losses
8p
17p
4q
6q
1p
0 1 2 3 4 5 6 7 8
Number of events
Driver arm gains
6p
1q
2p
11q
7p
20p
3q
16q
16p
11p
0 1 2 3 4 5
Number of events
Driver events of all classes
CARD11
PIM1
8p
KMT2D
B2M
MYD88
18
17p
CD79B
MYC
0 2 4 6 8
Number of events
